# Awareness, perception and factors affecting utilization of cervical cancer screening services among women in Ibadan, Nigeria: a qualitative study

**DOI:** 10.1186/1742-4755-9-11

**Published:** 2012-08-06

**Authors:** Chizoma Millicent Ndikom, Bola Abosede Ofi

**Affiliations:** 1 Department of Nursing, College of Medicine, University of Ibadan, Ibadan, Nigeria

**Keywords:** Awareness, Perception, Utilization, Cervical cancer screening, Women

## Abstract

**Background:**

Over the years awareness and uptake of cervical cancer screening services has remained poor in developing countries. Problems associated with cervical cancer incidence include late reporting, ignorance and cultural issues relating to cervical cancer screening. This study sought to explore the awareness, perception and utilization of cervical cancer screening among women in Ibadan as well as factors that influence utilization.

**Method:**

This is a qualitative study that utilized Eight Focus Group Discussions to collect information from women in selected health facilities in Ibadan, South West, Nigeria. The 82 participants were purposely recruited from women attending Antenatal clinics in 4 secondary and 4 primary health care facilities after approval was received from the Institutional Review Board in charge of the facilities. The focus group discussions were tape recorded and transcribed verbatim. The transcripts were analyzed into themes.

**Findings:**

The study provided qualitative information on the awareness, perception of the utilization of cervical cancer screening services among women in Ibadan. Participants were mainly married women (92.7%), mean age =27.6, SD =4.5, mainly traders (39%) and from Yoruba ethnic backgrounds (87.8%) and had secondary education (39%). The respondents reported not being aware of cervical cancer and were not utilizing the services. Though they did not know what cervical cancer screening entailed or the screening methods, they still believed that it is important since like for other diseases will help in early detection and treatment. The participants were eager to get more information from nurses on cervical cancer about cervical cancer screening. The major factors identified by the women that influence screening utilization were ignorance, Illiteracy, belief in not being at risk, having many contending issues, nonchalant attitude to their health, financial constraint and fear of having a positive result.

**Conclusion:**

There is an urgent need for more enlightenment about cervical cancer especially by health workers. Also, cervical cancer services should be made available at very affordable cost so that women can easily access the services in order to reduce incidence of invasive cancer.

## Background

Cancer of the cervix uteri is the second most common cancer among women worldwide [1-3]. with an estimated 529,409 new cases and 274,883 deaths in 2008. About 86% of the cases occur in developing countries, representing 13% of female cancers [1]. Each year approximately, 10,000 women develop cervical cancer, and about 8,000 women die from cervical cancer in Nigeria [4]. Spayne [5] reported in their study that over half the study population was under 50 years of age suggesting this disease is responsible for a disproportionately greater loss of life-years and social cost.” Records from cancer registry UCH indicate that the incidence is high; it was 353 out of 1942 total malignancies in 2007. Evidence of decline in incidence has been observed from countries like the United States where there are established screening protocols [6].

Sporadic screening is being carried out in Nigeria using opportunist method for those who visit certain clinics. Also, there is no standard policy or protocol for cervical cancer screening in Ibadan is similar to that of Nigeria which is sporadic or opportunistic;. It is more worrisome as all sexually active women are at risk for the development of cervical cancer. Where the services are available, many women seem not to be aware of the services. Services are mainly available in some secondary and tertiary health facilities at a cost that make it not accessible and affordable to many women.

Over the years awareness and uptake of services has remained poor despite all the studies on cervical cancer screening*.* Various studies indicate that cervical cancer screening services is poorly utilized and the awareness of the need for it is very low but can be treated if detected early [7-10]. Problems associated with cervical cancer incidence include late reporting, ignorance and cultural issues relating to cervical cancer screening (reference). . The barriers identified by Mutyaba ^3^ were “ignorance about cervical cancer, cultural constraint/beliefs about illness, economic factors, domestic gender power relations, alternative authoritative sources of reproductive health knowledge and unfriendly health care services”.

Women in developing countries like Nigeria seem to utilize reproductive health services more during pregnancy. They also use reproductive health services for post natal check up and family planning or when faced with various gynaecological problems. It is important to ensure that these women are screened in order reduce incidence of cervical cancer. Their visit to the clinics provides opportunity to give them information on the importance of the screening and where to get the services. The researchers observed that many women attending various health facilities have not been screened. Thus the need to explore the factors influencing utilization of cervical screening services among women in selected Health facilities in Ibadan, Oyo State.

Objectives of the study

1. To examine women’s awareness of cervical cancer

2. To investigate women’s perception of screening programmes

3. To evaluate the women’s utilization of screening services

4. To determine factors influencing utilization of services

5. To find ways of overcoming the problems

## Theoretical framework

### Health belief model

The health belief model is a psychosocial model proposed by Rosenstock (1966) in Stanhope and Lancaster [11] for studying and promoting the uptake of health services like screening. The model explains preventive behaviour.

The model assumes that belief and attitudes of people are critical determinants of their health-related actions. It holds that when cues to actions are present, the variations in uptake behaviour can be accounted for by beliefs concerning four sets of variables. These include:

· The individual’s view of own vulnerability to illness. If an individual does not see him or herself as being at risk of any problem, he or she will not seek care

· Belief about severity of the illness. The associated problem could be seen as little, therefore little attention will be required

· The person’s perception of the benefits associated with action to reduce the level of threat or vulnerability

· The individual’s evaluation of the potential barrier associated with the proposed action, this could be physical, psychological, financial and social.

### Framework of the Three Major Components of Health Belief Model

**Figure 1:**
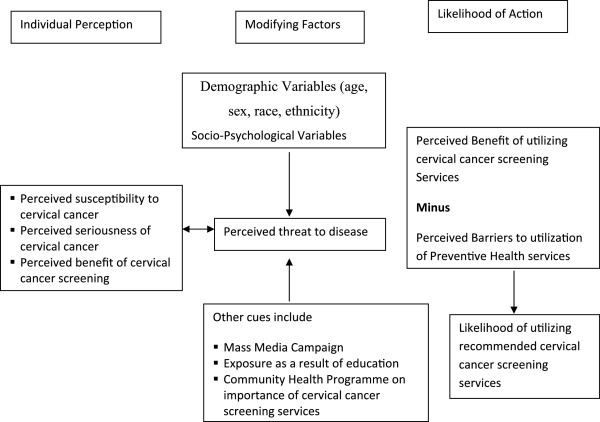
**Health belief Model Adapted from Stanhope and Lancaster**[11].

Figure 1 is used to illustrate the framework of the three major components of Health Belief Model as it relates to perception and utilization of cervical cancer screening services.

The three major components of the health belief model are: individual perception; modifying factors; and variables affecting likelihood of action:

· Individual perception: perception is the process of becoming aware of objects, qualities or relation by the way of sense organ. The individual’s perception of being at risk of cervical cancer will motivate the person to preventive services.

· Modifying factors: these are variables that change or improve likelihood of action. They include demographic variables, level of education, location of health facility, mass media etc. They affect perception of threat; increased knowledge will result in correct perception of threat based on scientific knowledge of cervical cancer.

· Likelihood of action: an individual will take action if he or she understands that there is a need and that the particular action will help in meeting the need. Also if barriers to the utilization of such services are minimized.

· Since cervical cancer is not usually noticed until late stage the call to go for screening seems to be ignored. Some women may not consider it as important because they have other competing needs. While others who may perceive screening as needful preventive health behaviour.

## Materials and methods

The study was carried out in eight selected health facilities in Ibadan, Oyo state. The choice of the health facilities was informed by the availability of MCH services and patient turnover. The health facilities consist of four (4) secondary health facilities and four (4) Primary Health Centres (PHCs). The 82 participants were purposely recruited from women attending the clinics. Antenatal clinic was used since both the primary and secondry health facility have it available with good attendance. Eighty (82) participants were purposely recruited for the study after approval was received from the Institutional Review Board in Oyo State Ministry of Health in charge of the facilities.

Data was collected through FGD; FGD was chosen as the method of Data collection as it provides real life data while discussing with the women. it gives room for more depth during information gathering. The group size was between 8 to 11 and eight sessions were conducted within 2 months each lasting around 60 minutes The 82 women participated in eight FGDs. The group Size was between 8 to11 each. Eight focus groups were carried out within two months, each lasting around 60 minutes. The researcher and three trained assistants were in the group as note taker, recorder and lead moderator.

The discussion was semi-structured using a Focus group discussion guide, and women were asked to talk freely on questions and issues raised during the discussion on cervical screening. Informed consent was obtained after study was explained to the participant. A structured questionnaire was used to collect their demographic data. The focus group discussions were tape recorded and transcribed verbatim. The transcripts were analyzed into themes. After familiarization with the transcripts, a thematic framework was developed with themes organized into 11 broad areas.

1. Common topics discussed by nurses in the clinic

2. The things you hear about cervical cancer outside the clinic

3. Perception about cervical cancer as an important topic during health talks

4. Information received about cervical cancer

5. Information received about screening methods

6. Perception about cervical cancer screening

7. Perceived role of cervical cancer screening in preventing death from the disease

8. Perception about early detection through the screening

9. Impression about the utilization of cervical cancer screening services by women

10. Perceived reasons for poor utilization of available cervical cancer screening services

11. Suggestions on ways that screening uptake can be improved

These were further grouped into four to meet the objectives of the study which include

· Awareness of cervical cancer screening methods

· Women’s Perception about Positive result at early stage of the disease

· Perceived Utilization of Cervical Cancer Screening Services

· Suggestions by the women on ways screening uptake can be improved

The discussion was carried out openly among the women as there was room for expression of views. Every participant was given a chance to respond to the questions to the best of their undestanding. All the responses were noted and organized into the themes. Many of the participants became aware of the disease (cervical cancer) during the course of the discussion.

## Results

### Socio-demographic characteristics of Participants

The demographic characteristics of each group are shown in Table 1. Women were mostly married (and aged between 26 and 30 years Mean =27.6, SD =4.5. They are mainly traders and from Yoruba ethnic backgrounds (87.8%) and had secondary education (39%). The majority were Muslims (58.5%). All the respondents were within the child bearing age, booked in ANC and have attended the clinic for more than once.

**Table 1:** Socio Demographic Characteristics of the

**Characteristics**	**Frequency n = 82**	**Percent (%)**
**Age(years)**	**Mean Age =27.6, SD =4.5**	
16–20	6	7.3
21–25	22	26.8
26–30	37	45.1
31–35	12	14.6
36-40	5	6.1
**Marital Status**		
Married	76	92.7
Single	1	1.2
Cohabiting	2	2.4
No response	3	3.7
**Occupation**		
Employed	16	19.5
Student	8	9.8
Civil servant	2	2.4
Unemployed	8	9.8
Artisan	14	17.1
Trading	32	39
No response	2	2.4
**Educational attainment**		
Primary	9	10.9
JSS	21	25.6
SSS	11	13.4
Poly technique	21	25.6
University	15	18.3
No response	5	6.1
**Religion**		
Christianity		
Islam	34	
48	41.5	
58.5		
**Ethnic group**		
Yoruba	72	87.8
Igbo	6	7.3
No response	4	4.8

### Common Topics during Health Talks

Table 2 presented Key topics discussed during health talks at the antenatal clinics as identified by women during FGDs. The findings show that cervical cancer screening was never a topic for discussion in the clinic. Some of the participants stated: 

"*We are told to take care of body, breast and private parts and under wears. Wear free clothes and not tight ones. Change twice daily, Bath twice daily. We are told not to be lazy because* we are *pregnant but carry out necessary house chores. Eat good food such as egg, vegetables, fish, liver and so on”.*

**Table 2:** Perceived utilization of cervical cancer screening services by women

**sn**	**Variable**	**Responses**
	▪ Perceived utilization of cervical cancer screening services by women	▪ Women are not utilizing it.
		▪ Only the elites utilize it
		▪ Common belief is that what you don’t know can’t kill you.
	▪ Perceived factors that influence uptake of cervical cancer screening services	▪ Lack of awareness of cervical cancer and facilities for screening
		▪ It is not important to them
		▪ nonchalant attitude to one’s health
		▪ Financial constraints may be the cause sometimes
		▪ Illiteracy, some people think that such services are for educated people
		▪ Belief that positive result is death warrant if they are tested positive so it is better not to go for screening
		▪ Screening services are not easily accessible
		▪ Poor information dissemination by health workers.

### Awareness and Common Information on Cervical Cancer

On Awareness of Cervical Cancer, majority of the participants stated that they have never heard about cervical cancer, only a few admitted the have heard about it. Some of the participants gave responses like this: 

“We don’t know about it; I have not heard about cancer of cervix; I only heard of Breast cancer; I am hearing it for the first time”.

Only a few women said they had heard about it and reported as follows:

“I heard it destroys the mouth of the womb and the person will not be able to deliver a baby and will eventually lead to the evacuation of the womb.; I heard it is a deteriorating disease, sometimes some babies would be delivered with mucous in the eyes, and it is dangerous especially for pregnant women.; I heard that it is not curable. It is linked with toilet diseases.”

The statements from those that have heard showed that they did not have accurate information about the disease.

### Cervical Cancer as an important Topic during Health Talk

On whether Cervical Cancer should be an important topic during health talk, all participants agreed that cervical cancer should be an important topic during health talks. They were very interested in receiving health talks on cervical cancer and they gave their reasons as follows: 

“It should be part of ANC information, so we can know about it since we don’t know much about it; We hear about cancer but we don’t know the meaning and what causes it; Should be discussed because it is very dangerous. It should be discussed because it is a disease of women, when we discuss it we know more about it, we know how to take care of ourselves; We believe that whatever we hear in the clinic are facts that we should know so that we will know how to take care of ourselves and prevent infections”.

### Women’s Awareness about cervical cancer screening

The participants did not know about cervical screening. Their responses were centered on the fact that they need more information. Common statements were; 

“No *idea; Good to know about it; It is good to have more enlightenment about cervical cancer; I think we need to know about the disease first before having saying what we feel about the screening; The government should be told so that they can make the screening tests available because it causes delay for some people in getting pregnant; It good as it makes us know if we are carrying the disease.*

### Awareness of cervical cancer screening methods

Majority have not heard about any screening method. Thus, they did not know about any type of screening methods. They made the following statements: 

“Not heard about any test; Don’t know; Have not really been taught; I just heard that one can go for scan. It is always being announced on the radio that women should go for screening but I have not heard of methods”.

### Women’s Perception about Positive result at early stage of the disease

Though the participants did not know much about cervical cancer but since it is a disease they believe it is better to treat early. They believe that cervical cancer like other cancers will get worse if not detected and treated early. 

“*Better to treat immediately to prevent complication; The only thing that will happen is that the person will first be treated to stop the spread of the disease; Prepare the mind of the person going for screening if not, the person may even die before the disease kills her; I don’t think they can manage cancer of the cervix and if it is detected early, the cervix of the person will have to be cut; When an early stage of the disease is detected, it now depends on the expertise of the health personnel on how to talk to people and give them hope”.*

Majority of the participants stated that early diagnosis and treatment will reduce death.

### Perceived Utilization of Cervical Cancer Screening Services

Table 2 shows statements about utilization of screening services. The women stated that the screening services are not really being utilized. Their statements were as follows*:*

“People have not been utilizing it; Will utilize services if asked to e.g. when asked to do tests we do it.; It is not common like HIV.; Only literates utilize screening services while illiterates usually feel that something you don’t know cannot kill you.; I think people will turn up if such programmes are organized for pregnant women; Most people in Africa are complacent about screening but the presentation of these screening services will determine the utilization of such services; I have not been to a hospital where they are doing it; I personally have not gone for such screening. It is possible to that most people have not been exposed to it so they have not been using it; I have not seen anyone use such service before”

Table 2 also provides a summary of the common the reasons for poor utilization of cervical cancer screening services. 

“It is due to nonchalant attitude to one’s health; Financial constraints may be the cause sometimes; Illiteracy, some people think that such services are for educated people Some people may think that it is death warrant if they are tested positive so it is better not to go for screening

There are few centres for screening; On the part of the health workers, I think the orientation about Cervical Cancer is low unlike HIV, nobody mentions it; Not heard about it; It should be introduced to the system so that whenever we come to the clinic, awareness will be created for people to know about it like HIV; some feel they can’t be affected; If it can be done for pregnant women so we can know our stand.

### Suggestions by the women on ways screening uptake can be improved

Table 3 provides summary of the suggestions made by the women. Most of their suggestions were centred on increasing awareness and making the services available. Some of their statements were: 

*“As HIV is being advertised on TV and radio, it should also be done for Cervical Cancer; It should be advertised on Radio so that people will go for screening and those who are positive will know about the disease and get treatment for it.; screening can be improved through orientation and awareness by nurses in streets and markets like immunization programmes; Information can be taken to schools and offices, hospitals and even to the market women. It should be explained to both uneducated and educated people; Education on cervical cancer should begin from nursery, primary and secondary.*"

"*Should be discussed at the clinics, posters should be made and public campaign should be done so that people can know about it.*

**Table 3:** Suggested measures for improving Screening uptake

	
· Suggestions on ways that screening uptake can be improved	· Increase Awareness of cancer of the cervix and screening through:
	* Media enlightenment as done for HIV it
	* Public awareness in the market places
	* Government should try to publicize the disease so that people will know about it.
	* Increased orientation and awareness by nurses in clinics, use of posters should be made and public campaign should be done so that people can know about it.
	* It should be explained to both uneducated and educated people
	* Education on cervical cancer should begin from nursery, primary and secondary.
	* It should be part of the topics discussed during ANC.
	· It should be included as part of the screening procedure for pregnant women when registering for ANC
	· Services should be available- clinics, house to house or community based service provision like done for immunization services
	· Should be made compulsory

## Discussion

The study provides information about awareness, attitudes and perception on cervical cancer and its screening methods.The study carried out among women in selected health facility in Ibadan has provided qualitative information on the perception of the utilization of cervical cancer screening among women. The demographic characteristics of each group are shown in Table 1. Women were mostly married (92.7%) and aged between 26 and 30 years (45.1%), mean =27.6, SD =4.5. Mainly traders (39%) and from Yoruba ethnic backgrounds (87.8%) and had secondary education (39%). The majority were Muslims (58.5%). All the respondents were within the child bearing age, booked in ANC and have attended the clinic for more than once. Generally the respondents reported not being aware of cervical cancer.

The study was summarized into the four objectives of the study.

### Common information women received about cervical cancer

In the study, women might not hear enough about cervical cancer in the clinics. The study has revealed that though many of these women had been to the clinic several times but have never been told about cervical cancer. Information gathered during the study showed that cervical cancer is not part of their routine health talks. This could be that other pregnancy related issues are discussed more in the clinic than cervical cancer. These women also utilize other MCH services as this was not their first pregnancies. The women were more informed about HIV screening which is done routinely for every consenting pregnant woman as a national policy in Nigeria.

### Awareness of women about cervical cancer screening

Though they did not know what screening entails but the women believe that screening is good as it will help those that have problem to know early so they can be treated. Poor knowledge of cervical cancer among women has been reported in various studies also^7,8, 8,9,10^. A study of influences on uptake of reproductive health services revealed that “knowledge about cervical cancer among the women was very low” ^3^. Prominent in their finding was the fact that patients are not given adequate information on cervical cancer and screening^3^. This shows that women are willing to know about their health but nurses are not using their vantage positions to provide necessary information on cervical cancer.

### Utilization of cervical cancer screening services among the women

The utilization of screening services as the women stated that it is not possible to use what they don’t know about. Women were not using the services as they did not know about the services or where to obtain such services. “Cervical cancer screening based on cytologic examination is largely unavailable in developing countries or made available to a small, select group of women in private facilities, maternal child health sites, or family-planning clinics, missing the age groups at highest risk for precancerous lesions”^7^. According to Ayinde and Omigbodun^8^ 93.2% of respondents in their study have never had Pap smears performed. Only 14.1% of respondents had pap smear in another study and there was a significant variation in utilization of Pap test (Pearson Chi-square 14.67, *P* < 0.05), and a significant correlation between Pap smear awareness and utilization (*P* < 0.001).^9^.

### Factors that influence uptake of cervical cancer screening

The major factors identified by the women in the study are lack of awareness about the screening. Illiteracy, some people think that such services are for educated people. Also, the facts that when people are healthy they don’t bother about preventive services as they have other contending problems. It is seen as generally not important and many nonchalant attitude to their health. Financial constraint is another problem as the available services are not free. The poverty level in our society is quite high.

Some people may think that it is death warrant if they are tested positive so it is better not to go for screening. Screening services were not available in most of the centres.

This finding is supported by various studies. A Swedish Study reported that non attendance to cervical screening was positively associated with time-consuming and economic barriers [12]. Time is a problem because women have so many responsibilities e.g. thus cervical cancer screening could be given less priority in demanding real life settings [12]. Most screening programmes rely on Pap smear which are are complex and costly to run especially in developing countries where health systems and infrastructures are weak [13]. Also, poor knowledge, underlying health and cultural beliefs, attitudes, language and unhelpful attitudes of health professionals are important barriers [14]. Other Barriers to screening include increased age, nonwhite race/ethnicity, low educational level, low income, decreased access, in sufficient funding, and unfavourable attitudes towards screenin [15]. Similarly, the barriers identified by Mutyaba ^3^ were “ignorance about cervical cancer, cultural constraint/beliefs about illness, economic factors, domestic gender power relations, alternative authoritative sources of reproductive health knowledge and unfriendly health care services”. Also, lack is cervical cancer control programme could also be a factor influencing utilization of services [16].

· In line with the health belief model , the study has shown that the women were not really aware of cervical cancer nor understood their susceptibility to the disease thus they were not motivated to utilize cervical screening services. Since the women were not well educatedand and the screening facility was not readily available and other barriers identified. It is not suprising that these women were not able to utilize cervical cancer screening services.

### Limitation of the study

The study was carried out in antenatal clinic where a good number of women within the target group could be accessed for information. Though it provides access to the target women it did not cover information from women using other Maternal and child health services.

Suggestions by the women on ways screening uptake can be improved

Women have still re-echoed the need to expedite effort in increasing awareness about cervical cancer. This fact cannot be over emphasized as most studies already presented showed that awareness and knowledge of cervical cancer is low. Increaed information disamination and couselling will help to protect some rights of the women [17]. Non availability of screening services is another major challenge the participants want to be addressed urgently. They also suggest out reach programmes in various communities.

## Conclusion

This study has shown that the awareness of cervical cancer screening was very low among women utilizing selected health facilities. It could be worse among those not coming to the health facilities. They claimed that information on cervical cancer is not being provided by health workers. The participants were eager to know more about cervical cancer and screening methods. A major barrier to majority is the unavailability of screening services in most of facilities. Thus there is urgent need to improve awareness as well as provide affordable cervical cancer screening services. Nurses need to include cervical cancer as one of the topics to be discussed in the clinics as it is a very important reproductive health issue. It is also important to develop policy on cervical cancer screening as a nation.

## Competing interests

The authors declare that they have no competing interest.

## Author’s contributions

CMN and BAO Jointly designed the study while CMN carried it out under supervision of BAO. CMN drafted the manuscript with contributions from BAO. All authors read and approved the final manuscript.
